# COVID-19 infection is associated with anti-M alloantibody development in pediatric patients: immunological characteristics and clinical implications for transfusion safety

**DOI:** 10.1186/s13052-026-02258-x

**Published:** 2026-04-24

**Authors:** Ming-Wei Yin, Xue-Qi Peng, Ji-Hua Ma, Gui-Zhen Jiang, Ning Zhao, Xin-Yu Huang, Si Shi, Xue-Jun Chen

**Affiliations:** 1 Department of Blood Transfusion, Children’s Hospital, Zhejiang University School of Medicine, National Clinical Research Center for Children and Adolescents’ Health and Diseases, No. 3333 Binsheng Road, Binjiang District, Hangzhou, 310052 China; 2 Department of Clinical Laboratory, Children’s Hospital, Zhejiang University School of Medicine, National Clinical Research Center for Children and Adolescents’ Health and Diseases, Hangzhou, Zhejiang Province China; 3 Institute of Transfusion Medicine, Blood Center of Zhejiang Province, Hangzhou, Zhejiang Province China

**Keywords:** Anti-M, COVID-19, Alloantibody, Pediatric, Transfusion safety, Molecular mimicry

## Abstract

**Background:**

Anti-M antibodies are commonly observed as naturally occurring antibodies in the MNS blood group system, and their production has been linked to various microbial infections through molecular mimicry. However, the association between COVID-19 infection and anti-M antibody development in pediatric patients remains unexplored.

**Methods:**

This retrospective matched case-control study included 31 anti-M positive pediatric patients and 31 matched anti-M negative controls with NN phenotype from October 2022 to May 2025. Anti-M isotypes (IgM/IgG) were distinguished using saline and dithiothreitol-preserved plasma methods, followed by titer determination and IgG subclass analysis. Plasma cytokines were quantified by flow cytometry. COVID-19 infection history within 2 years was recorded.

**Results:**

Anti-M positive patients had significantly higher COVID-19 infection rates than controls (54.84% vs. 19.35%, OR = 5.06, *P* = 0.008); this association remained significant after adjustment for age and sex (adjusted OR = 5.35, 95% CI: 1.67–17.17, *P* = 0.005). Among those with IgG antibodies, anti-M IgG titers inversely correlated with the interval since COVID-19 infection (*r* = -0.63, *P* = 0.008). Reactive IgG (IgG1/IgG3) exhibited significantly higher titers than inactive IgG (median 64 vs. 4, *P* < 0.001), with all high-titer cases (≥ 64) exclusively in the reactive group (11/31, 35.48%). Anti-M positive patients showed elevated IL-8 (*P* = 0.006) and IL-12p70 (*P* = 0.029), and unexpectedly lower bilirubin levels (*P* < 0.01), suggesting metabolic adaptation rather than hemolysis.

**Conclusion:**

COVID-19 infection is significantly associated with anti-M alloantibody development in NN-phenotype children, with over one-third reaching clinically significant titers. These findings suggest that enhanced antibody screening should be considered for pediatric transfusion candidates with recent viral infections, although the single-center design with modest sample size (31 pairs) warrants multi-center validation.

**Supplementary Information:**

The online version contains supplementary material available at 10.1186/s13052-026-02258-x.

## Highlights

1. COVID-19 infection is significantly associated with anti-M antibody development in NN-phenotype children (54.84% vs. 19.35%, OR = 5.06, *P* = 0.008).

2. Anti-M IgG titers inversely correlate with the interval since COVID-19 infection (*r* = -0.63, *P* = 0.008), consistent with an infection-triggered mechanism potentially through molecular mimicry.

3. Over one-third (35.48%) of anti-M positive children developed high-titer (≥ 64) reactive IgG antibodies (IgG1/IgG3 positive) with potential for hemolytic transfusion reactions.

4. Anti-M positive patients exhibited elevated IL-8 and IL-12p70, indicating a distinct pro-inflammatory signature associated with antibody production.

5. Enhanced antibody screening should be considered for pediatric transfusion candidates with recent viral infections, with IgG subclass determination for risk stratification, pending validation in prospective studies.

**Supplementary Information:**

The online version contains supplementary material available at 10.1186/s13052-026-02258-x.

## Graphical Abstract

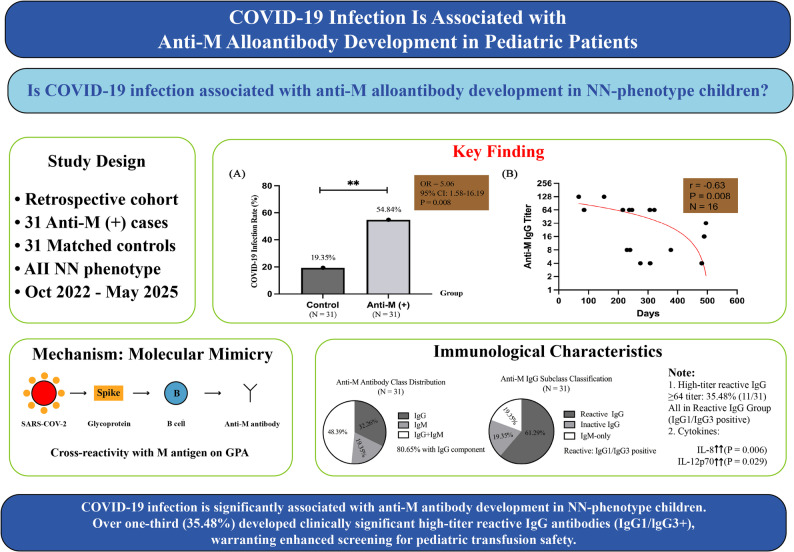

## Introduction

Anti-M antibody is one of the most commonly detected alloantibodies in the MNS blood group system. While often observed as a naturally occurring antibody with limited clinical significance [1], anti-M can become clinically relevant when present as IgG class antibodies, particularly IgG1 and IgG3 subclasses that are capable of activating complement and mediating hemolytic reactions [1, 2]. High-titer IgG anti-M (≥ 64) has been associated with acute or delayed hemolytic transfusion reactions and hemolytic disease of the fetus and newborn (HDFN) [3, 4]. The production of naturally occurring anti-M antibodies has long been recognized to be associated with microbial infections through molecular mimicry mechanisms [5]. Kao et al. first reported in 1978 that children with acute bacterial infections caused by *Haemophilus influenzae*, *Proteus mirabilis*, *Staphylococcus aureus*, and *Neisseria meningitidis* could develop anti-M antibodies [6]. The M antigen, carried by glycophorin A (GPA), differs from the N antigen by only two amino acids (S1L, G5E), and its epitope structure may share similarities with certain pathogen surface antigens, thereby triggering cross-reactive B cell activation [7].

Severe acute respiratory syndrome coronavirus 2 (SARS-CoV-2), the causative agent of COVID-19, has been shown to induce various autoantibodies and alloantibodies through immune dysregulation [8, 9]. In 2022, Jeannet et al. reported the first case of de novo anti-M alloantibody emergence in a severe COVID-19 patient, demonstrating an unmutated IgM lambda antibody likely produced through extrafollicular B cell activation [10]. This case suggested that SARS-CoV-2 infection could trigger anti-M production, but systematic studies investigating this association in pediatric populations are lacking.

During the COVID-19 pandemic and the subsequent endemic period, we observed an apparent increase in anti-M positive cases among pediatric patients at our center. This clinical observation prompted us to systematically investigate whether COVID-19 infection is associated with anti-M antibody development in children. To address this question, we designed a retrospective matched case-control study comparing anti-M positive children with matched anti-M negative controls, all of NN phenotype (lacking M antigen), to characterize the immunological features of pediatric anti-M antibodies and explore their association with COVID-19 infection and inflammatory status.

## Materials and methods

### Participants

This retrospective matched case-control study was conducted at a single pediatric center. From October 2022 to May 2025, we enrolled 31 pediatric patients with positive anti-M antibodies as the observation group. All patients had the NN phenotype in the MNS blood group system and no history of blood transfusion. Cases of neonatal hemolytic disease of the MNS system were excluded. For the control group, we identified candidates from same-period anti-M antibody-negative samples by performing MN phenotyping using commercial anti-M and anti-N reagents to select NN-phenotype individuals. Controls were then matched 1:1 to cases using propensity score matching (PSM) performed in SPSS. The propensity score was estimated using logistic regression with age, sex, and primary diagnosis category as covariates. Nearest-neighbor matching without replacement was performed with a caliper of 0.2 standard deviations of the logit of the propensity score.

### Definition of COVID-19 infection history

Based on the reported duration of anti-M antibody persistence [11], we set the threshold for COVID-19 infection history at 2 years. COVID-19 infection history was ascertained through structured telephone follow-up interviews with parents/guardians. COVID-19 diagnoses were based on: (1) positive SARS-CoV-2 nucleic acid testing (RT-PCR) from hospital or community testing sites; (2) positive rapid antigen test results; or (3) physician-diagnosed COVID-19 based on clinical presentation during confirmed community outbreaks (particularly during the December 2022 - February 2023 Omicron wave in China, when testing capacity was overwhelmed and clinical diagnosis was widely practiced). Patients with reported COVID-19 infection within 2 years before inclusion were classified as COVID-19 positive; those with infection more than 2 years prior or no reported infection were classified as COVID-19 negative. The interval between COVID-19 infection and anti-M detection was recorded in days. Other pathogen infections were based on laboratory tests performed during the current hospital visit.

### Blood sample collection

Residual ethylenediaminetetraacetic acid (EDTA) anticoagulated whole blood and clotted blood samples were collected following clinical testing. EDTA samples were centrifuged (1,000 × g for 5 min at room temperature) to separate plasma and packed red blood cells (RBCs). Clotted blood samples were centrifuged under identical conditions to harvest serum. All derived components (plasma, RBCs, serum) were immediately aliquoted and stored at -20 °C for less than 48 h, then subsequently transferred to -80 °C for long-term preservation until analysis.

### MNS blood group serological typing

Patient RBCs were washed and suspended to 2–5% in saline, then tested against monoclonal anti-M and anti-N antibodies using standard tube agglutination. Equal volumes of antiserum and RBCs suspension were incubated at room temperature for 15 min. Tubes were centrifuged (1000 × g, 15 s), and agglutination was visually assessed after gentle resuspension. Known positive and negative controls were included with each antiserum batch to validate procedures. Agglutination indicated antigen presence; smooth resuspension indicated absence. All steps followed manufacturer instructions.

### Anti-M antibody typing and titer determination

Serum samples were tested for the presence and titer of anti-M IgM and IgG antibodies. IgM were determined using the saline tube technique at room temperature with serial dilutions. The IgM titer was defined as the reciprocal of the highest dilution yielding ≥ 1 + agglutination. For IgG determination, serum was pretreated with an equal volume of 0.01 M dithiothreitol (DTT) at 37 °C for 40 min to inactivate IgM, then tested using the indirect antiglobulin test (IAT) in microcolumn gel cards (DiaMed anti-human globulin cards, anti-IgG specific). IgG titer was the reciprocal of the highest DTT-treated dilution yielding a positive reaction (≥ 1+). IgM and IgG anti-D antibodies were used as controls to validate the effectiveness of DTT.

### Detection of IgG antibody subtypes

Serum samples were tested for IgG1 and IgG3 subclasses using a commercial microcolumn gel assay (Human IgG1/IgG3 Antibody Detection Card, Changchun BoXun Biotechnology Co., Ltd.). Each column contains gel coupled with murine monoclonal anti-human IgG1 (clone 3C11), IgG3 (clone 7C11), or polyclonal goat anti-human IgG (AHG). Testing was performed according to the manufacturer’s instructions. Briefly, a suspension of reagent RBCs sensitized with the test antibody was applied to each microcolumn, incubated at 37 °C for 15 min, and then centrifuged.

### Cytokine quantification by flow cytometry

Cytokine analysis was performed on residual plasma samples when sufficient volume (≥ 200 µL) remained after completion of all routine clinical testing. The availability of cytokine data was determined by residual sample volume rather than patient selection criteria, resulting in a subgroup of 30 patients (observation *N* = 18, control *N* = 12) with complete cytokine measurements. Plasma concentrations of human IL-1β, IL-2, IL-4, IL-5, IL-6, IL-8, IL-10, IL-12p70, IL-17, TNF-α, IFN-α, and IFN-γ were simultaneously quantified using the CECER Multi-Cytokine Detection Kit (Immunofluorescence Method) by flow cytometry, according to the manufacturer’s instructions. Briefly, samples were incubated with antibody-conjugated capture beads conjugated with monoclonal antibodies specific to each cytokine. Following washing, biotinylated detection antibodies were added, followed by streptavidin-phycoerythrin (PE). Cytokine concentrations were determined by interpolating sample fluorescence intensities (MFI) against a standard curve generated from kit-provided recombinant cytokine standards run concurrently. Samples falling outside the assay range were diluted and reanalyzed.

### Quantification of immunoglobulins and complements

Serum concentrations of total IgE, IgG, IgA, IgM, complement C3 and C4 were measured using automated platforms with manufacturer-matched reagents. Serum IgE levels were quantified using the Siemens BN II System nephelometer (Siemens Healthcare Diagnostics Inc.) via latex-enhanced immunonephelometry. Briefly, samples were diluted and incubated with anti-human IgE antibody-coated latex particles, and scattered light intensity was measured at 840 nm. Manufacturer-specific calibrators and controls were used. Concentrations of IgG, IgA, IgM, C3, and C4 were determined using the Hitachi 7180 automated biochemistry analyzer (Hitachi High-Tech Corporation) via immunoturbidimetry. Samples were incubated with kit-specific polyclonal antisera, and turbidity was measured photometrically.

### Statistical analysis

The normality of continuous variable distributions was assessed using the Shapiro-Wilk test. Variables with *P* > 0.05 in both groups were considered normally distributed and compared using independent-sample t-tests, with results presented as mean ± standard deviation (SD). Variables failing the normality assumption in either group were compared using Mann-Whitney U tests (two-group comparisons) or Kruskal-Wallis tests (three-group comparisons), with results presented as median (interquartile range, IQR). Chi-squared and Fisher’s exact tests were used for categorical variables. Pearson correlation analysis was used to examine the relationship between two continuous variables. PSM was performed as described in the Participants section. Multivariable logistic regression was performed with anti-M antibody status as the dependent variable and COVID-19 infection history as the primary predictor, adjusted for age (continuous, in months) and sex (binary). Adjusted odds ratios (aOR) with 95% confidence intervals (CI) were reported. Post-hoc power analysis was conducted to evaluate the study’s ability to detect the observed effects. For the primary outcome (the association between COVID-19 infection and anti-M antibody positivity), the achieved power was calculated using a two-proportion z-test with α = 0.05 (two-sided). Effect sizes were reported as Cohen’s h for proportion comparisons, odds ratios with 95% CI for categorical outcomes, and rank-biserial correlation coefficient (r) for non-parametric comparisons. Statistical analysis was performed using SPSS software, version 20.0 (SPSS, Chicago, IL, USA). Effect size calculations and post-hoc power analysis were performed using Python 3.10 with SciPy and StatsModels packages. *P* < 0.05 was considered statistically significant.

## Results

### Result 1. Baseline characteristics of the study population

Table 1 summarizes the biological and laboratory parameters of the cohort (*N* = 62), comparing control (*N* = 31) and observation (*N* = 31) groups. The median age was 39.0 months (IQR 55.0), with 28 females (45.16%), and exhibited comparable demographic distributions across age (*P* = 0.858) and sex (*P* = 0.799). Blood type frequencies (A: 35.48%, B: 27.42%, O: 29.03%, AB: 8.06%) and clinical diagnoses (tonsillar/adenoidal hypertrophy: 22.58%, other inflammatory conditions: 20.97%, and surgical diseases: 56.45%) did not differ significantly (*P* = 0.189 and *P* = 0.953, respectively). In laboratory tests, the observation group had higher platelet counts ((341.9 ± 87.0) vs. (302.6 ± 60.8) ×10⁹/L, *P* = 0.043) but lower levels of total bilirubin (5.75 vs. 9.20 µmol/L, *P* = 0.001, *r* = 0.49), direct bilirubin (1.45 vs. 2.50 µmol/L, *P* = 0.001, *r* = 0.50), and indirect bilirubin (4.35 vs. 6.40 µmol/L, *P* = 0.004, *r* = 0.45) compared to controls, with medium-to-large effect sizes. No significant differences were observed in C-reactive protein (CRP), hemoglobin, white blood cells (WBC) count, or neutrophil ratio (all *P* > 0.05).

**Table 1:** Baseline characteristics of the study population

Characteristics	All (*N* = 62)	Control (*N* = 31)	Observation (*N* = 31)	*P* value
**Demographics**				
Age (months), Median (IQR)	39.0 (55.0)	37.0 (52.0)	40.0 (56.0)	0.858
Female, N (%)	28 (45.16)	13 (41.94)	15 (48.39)	0.799
**ABO Blood Type**,** N (%)**				
A	22 (35.48)	8 (25.81)	14 (45.16)	0.189
B	17 (27.42)	8 (25.81)	9 (29.03)	
O	18 (29.03)	12 (38.71)	6 (19.35)	
AB	5 (8.06)	3 (9.68)	2 (6.45)	
**Primary Diagnosis**,** N (%)**				
Tonsillar/adenoidal hypertrophy	14 (22.58)	7 (22.58)	7 (22.58)	0.953
Other inflammatory diseases	13 (20.97)	7 (22.58)	6 (19.35)	
Surgical disease	35 (56.45)	17 (54.84)	18 (58.06)	
**Laboratory Tests**				
Platelet (×10⁹/L), Mean ± SD	322.3 ± 77.0	302.6 ± 60.8	341.9 ± 87.0	0.043*
CRP (mg/L), Median (IQR)	0.36 (1.93)	0.39 (1.54)	0.36 (2.67)	0.864
Hemoglobin (g/L), Median (IQR)	123 (12)	123 (8)	122 (15)	0.493
WBC (×10⁹/L), Median (IQR)	7.73 (4.20)	7.87 (4.11)	7.56 (4.28)	0.751
Neutrophil ratio (%), Mean ± SD	47.3 ± 17.1	49.1 ± 18.4	45.5 ± 15.9	0.725
Total bilirubin (µmol/L), Median (IQR)	7.50 (4.65)	9.20 (3.88)	5.75 (4.60)	0.001**
Direct bilirubin (µmol/L), Median (IQR)	2.00 (1.65)	2.50 (1.40)	1.45 (1.00)	0.001**
Indirect bilirubin (µmol/L), Median (IQR)	5.30 (3.40)	6.40 (2.85)	4.35 (3.40)	0.004**

Continuous variables were tested for normality using the Shapiro-Wilk test. Normally distributed variables are presented as mean ± SD (independent-sample t-test); non-normally distributed variables as median (IQR) (Mann-Whitney U test). IQR, interquartile range; SD, standard deviation; CRP, C-reactive protein; WBC, white blood cell. *P < 0.05; **P < 0.01

### Result 2. The production of anti-M antibodies is associated with COVID-19 infection

**Fig. 1:**
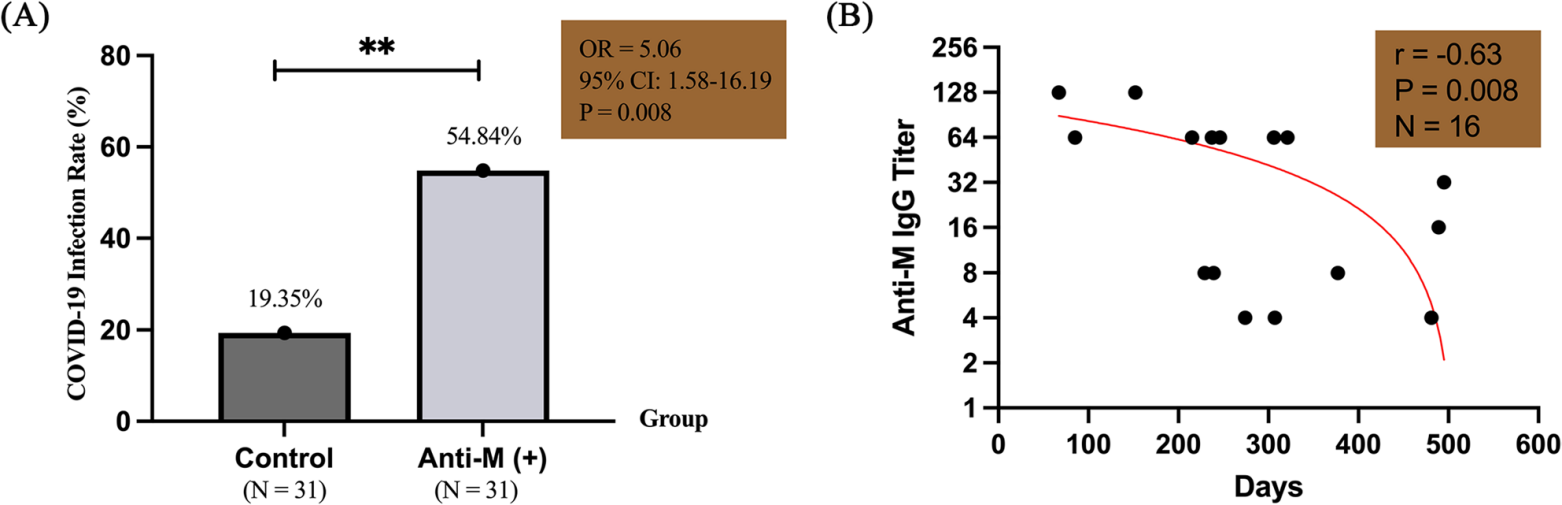
Association between COVID-19 infection and anti-M antibody production. (**A**) Bar chart comparing COVID-19 infection rates (within 2 years) between anti-M positive (observation, *N* = 31) and anti-M negative (control, *N* = 31) groups. (**B**) Scatter plot of anti-M IgG titers versus interval (days) since COVID-19 infection in patients with reported COVID-19 history and detectable IgG antibodies (*n* = 16). Pearson correlation coefficient *r* = -0.63, *P* = 0.008

Pathogen profiling revealed significantly higher COVID-19 infection rates (within 2 years) in anti-M positive patients compared to controls (54.84% [17/31] vs. 19.35% [6/31], OR = 5.06, 95% CI: 1.58–16.19, *P* = 0.008), with anti-M positive patients exhibiting substantially fewer infection-free cases (35.48% vs. 87.10%, *P* < 0.001) and increased viral co-infections (45.16% vs. 6.45%) (Table 2; Fig. 1A). While bacterial infection rates were identical between groups (6.45% each), other pathogen infections (primarily *Streptococcus pneumoniae*) occurred more frequently in the anti-M positive cohort (12.90% vs. 0%, 4/31).

**Table 2:** Comparison of COVID-19 and other pathogen infections between groups

Characteristics	Control (*N* = 31)	Observation (*N* = 31)	OR (95% CI)	*P* value
**COVID-19 History (within 2 years),** ***N (%)***				
Yes	6 (19.35)	17 (54.84)	5.06 (1.58–16.19)	0.008**
No	25 (80.65)	14 (45.16)		
**Current Pathogen Status**,** N (%)**				
None	27 (87.10)	11 (35.48)		< 0.001***
Virus	2 (6.45)	14 (45.16)		
Bacteria	2 (6.45)	2 (6.45)		
Others	0 (0)	4 (12.90)		

Current Pathogen Status are primarily categorized into no infection, viral infections (including adenovirus, rhinovirus, human herpesvirus 1, respiratory syncytial virus, and cytomegalovirus), bacterial infections, and other pathogen infections (mainly Streptococcus pneumoniae). Fisher’s exact test was used for COVID-19 history comparison; Chi-squared test for current pathogen status. CI, confidence interval; OR, odds ratio. **P < 0.01; ***P < 0.001

Serological analysis demonstrated a significant inverse correlation between anti-M IgG titers and interval since COVID-19 infection (*r* = -0.63, *P* = 0.008, *n* = 16), indicating higher titers proximal to viral exposure (Fig. 1B). Among the 17 COVID-19 positive patients in the observation group, 16 had detectable IgG anti-M antibodies. This temporal pattern is consistent with an infection-triggered immune response, with antibody levels declining progressively as time from infection increases, although a definitive causal relationship cannot be established from this observational design.

Multivariable logistic regression confirmed that COVID-19 infection history remained a significant predictor of anti-M antibody positivity after adjusting for age and sex (adjusted OR = 5.35, 95% CI: 1.67–17.17, *P* = 0.005), while neither age (aOR = 1.00, 95% CI: 0.98–1.01, *P* = 0.715) nor sex (aOR = 0.71, 95% CI: 0.24–2.10, *P* = 0.535) was independently associated with anti-M status, consistent with the matched design. Post-hoc power analysis indicated that the study had 82.4% power to detect the observed association between COVID-19 infection and anti-M antibody positivity (Cohen’s h = 0.76) at α = 0.05.

### Result 3. Immunological characteristics of anti-M antibodies in 31 cases of anti-M positive patients

**Fig. 2:**
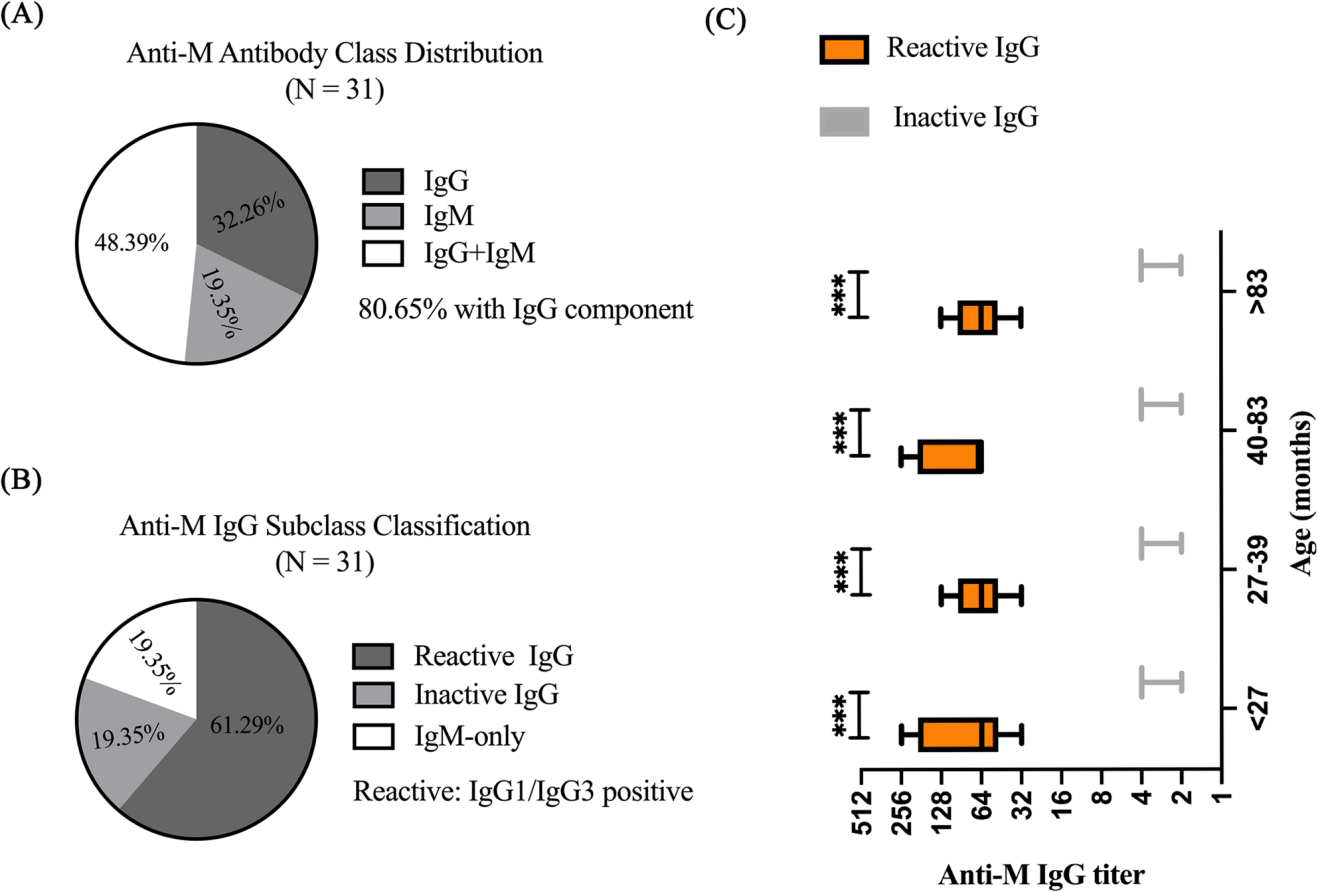
Immunological characteristics of anti-M antibodies in the observation group. (**A**) Pie chart showing the distribution of anti-M antibody classes among 31 anti-M positive patients: IgG-only (32.26%), IgM-only (19.35%), and IgG + IgM (48.39%), with 80.65% having an IgG component. (**B**) Pie chart showing the classification of 31 anti-M positive patients by IgG subclass reactivity: reactive IgG (IgG1 and/or IgG3 positive, 61.29%, *N* = 19), inactive IgG (IgG1 and IgG3 negative, 19.35%, *N* = 6), and IgM-only (19.35%, *N* = 6). (**C**) Age-stratified analysis of IgG titers between reactive and inactive IgG groups across four age quartiles (< 27, 27–39, 40–83, > 83 months; all *P* < 0.001)

As shown in Fig. 2A, anti-M isotype distribution revealed IgG-only (32.26%, 10/31), IgM-only (19.35%, 6/31), and IgG + IgM (48.39%, 15/31) profiles, with a total of 80.65% (25/31) having IgG antibodies. According to the type of antibody response, 31 children were divided into reactive IgG (IgG1 and/or IgG3 positive, *N* = 19), inactive IgG (IgG1 and IgG3 negative, *N* = 6), and IgM-only (*N* = 6) groups (Fig. 2B). The reactive IgG group showed significantly higher IgG titers than inactive IgG, representing a 16-fold increase (median 64 vs. 4, *P* < 0.001), with all high-titer cases (IgG ≥ 64, *n* = 11, 35.48%) exclusively in this group (Table 3).

**Table 3:** Immunological characteristics of anti-M antibodies in the observation group

Characteristics	All (*N* = 31)	Reactive IgG (*N* = 19)	Inactive IgG (*N* = 6)	IgM-only (*N* = 6)	*P* value
Age (months), Median (IQR)	40 (56)	41 (67)	53 (35)	30 (38)	0.672
IgM titer, Median (IQR)	2 (4)	2 (4)	0 (3)	4 (30)	0.089
IgG titer, Median (IQR)	16 (62)	64 (120)	4 (0)	0 (0)	< 0.001***
**Antibody Class**,** N (%)**					
IgG only	10 (32.26)	6 (31.58)	4 (66.67)	0 (0)	< 0.001***
IgM + IgG	15 (48.39)	13 (68.42)	2 (33.33)	0 (0)	
IgM only	6 (19.35)	0 (0)	0 (0)	6 (100)	
**High-titer IgG (≥ 64)**,** N (%)**	11 (35.48)	11 (57.89)	0 (0)	0 (0)	0.001**

Reactive IgG: IgG1 and/or IgG3 positive; Inactive IgG: IgG1 and IgG3 negative. Continuous variables are presented as median (IQR) and compared using Kruskal-Wallis test; categorical variables compared using Fisher’s exact test. IQR, interquartile range. **P < 0.01; ***P < 0.001

Age-based subgroup analyses consistently demonstrated higher IgG titers in reactive versus inactive groups across quartiles (< 27 months, 27–39 months, 40–83 months, > 83 months; all *P* < 0.001), though no inter-subgroup differences emerged within the reactive IgG group (*P* = 0.453) (Fig. 2C). The consistency of higher IgG titers in reactive versus inactive groups across all age quartiles with no significant variation within the reactive group indicates that the pathogenic potential of reactive IgG anti-M antibodies is independent of patient age. This age-independent pattern supports an infection-triggered mechanism rather than an age-dependent developmental process. Antibody class distribution differed significantly across three groups (*P* < 0.001): the reactive IgG group primarily exhibited IgG + IgM (68.42% vs. 33.33% in inactive IgG), whereas the inactive group was predominantly IgG-only (66.67% vs. 31.58% in reactive IgG). No significant intergroup differences existed in age (*P* = 0.672). IgM titers trended higher in the IgM-only group (median 4) versus reactive IgG (median 2) and inactive IgG (median 0), though not statistically significant (*P* = 0.089).

Notably, none of the anti-M positive patients experienced hemolytic transfusion reactions during the study period, as all antibodies were identified during pre-surgical antibody screening, and M-antigen negative blood was prospectively arranged when transfusion was indicated.

### Result 4. IL-8 and IL-12p70 levels were higher in anti-M positive patients compared to negative patients

**Fig. 3:**
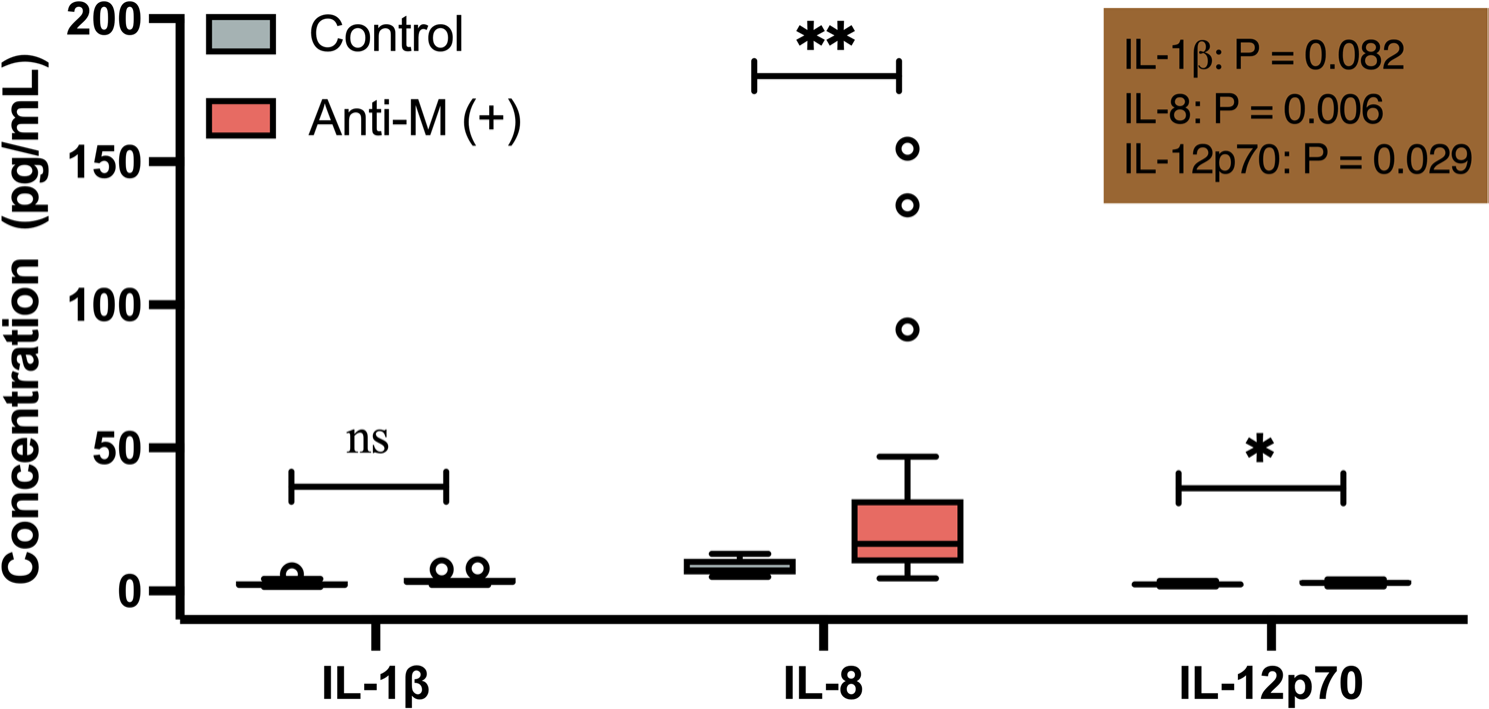
Plasma cytokine levels in anti-M positive and negative groups. Box plots comparing concentrations of IL-1β, IL-8, and IL-12p70 between control (*N* = 12) and observation (*N* = 18) groups. Boxes represent the interquartile range (IQR), horizontal lines within boxes indicate medians, whiskers extend to minimum and maximum values, and circles represent outliers. ns, not significant; *P < 0.05; **P < 0.01

Cytokine analysis was available for a subgroup of 30 patients (observation *N* = 18, control *N* = 12) determined by residual plasma volume availability (see Methods). Comparison of key baseline characteristics between cytokine-tested and non-tested subgroups revealed no significant differences in age, sex, or COVID-19 infection history (all *P* > 0.05; Supplementary Table S1). As shown in Table 4, comparative cytokine analysis revealed significant pro-inflammatory activation in anti-M positive patients versus negative controls, with markedly higher IL-8 (median 16.34 (17.13) vs. 7.25 (5.30) pg/mL, *P* = 0.006, *r* = 0.60) and increased IL-12p70 (median 2.69 (1.00) vs. 2.35 (0.72) pg/mL, *P* = 0.029, *r* = 0.48) (Fig. 3). IL-1β showed a trend toward elevation (median 3.12 (1.46) vs. 2.20 (1.32) pg/mL, *P* = 0.082, *r* = 0.38), though not reaching statistical significance, which may reflect limited power in this subgroup analysis (*N* = 30). No significant differences were observed in Th1/Th2 cytokines (IL-2, *P* = 0.241; IL-4, *P* = 0.489; IFN-γ, *P* = 0.325), innate immunity markers (IL-6, *P* = 0.258; TNF-α, *P* = 0.933; IFN-α, *P* = 0.722), or regulatory/Th17 cytokines (IL-5, *P* = 0.850; IL-10, *P* = 0.675; IL-17, *P* = 0.258).

**Table 4:** Comparison of plasma cytokines between groups

Cytokine	Control (*N* = 12)	Observation (*N* = 18)	*P* value
IL-1β (pg/mL), Median (IQR)	2.20 (1.32)	3.12 (1.46)	0.082
IL-2 (pg/mL), Median (IQR)	2.80 (0.85)	3.20 (0.98)	0.241
IL-4 (pg/mL), Median (IQR)	2.70 (0.75)	2.89 (0.79)	0.489
IL-5 (pg/mL), Median (IQR)	1.00 (0.50)	1.04 (0.28)	0.850
IL-6 (pg/mL), Median (IQR)	3.00 (3.90)	4.03 (4.00)	0.258
IL-8 (pg/mL), Median (IQR)	7.25 (5.30)	16.34 (17.13)	0.006**
IL-10 (pg/mL), Median (IQR)	5.40 (4.60)	4.92 (3.08)	0.675
IL-12p70 (pg/mL), Median (IQR)	2.35 (0.72)	2.69 (1.00)	0.029*
IL-17 (pg/mL), Median (IQR)	6.50 (3.70)	7.56 (3.31)	0.258
TNF-α (pg/mL), Median (IQR)	2.20 (1.01)	2.21 (1.19)	0.933
IFN-α (pg/mL), Median (IQR)	0.79 (0.43)	0.73 (0.63)	0.722
IFN-γ (pg/mL), Median (IQR)	3.10 (2.10)	2.95 (1.28)	0.325

Values are presented as median (IQR). Mann-Whitney U test was used for all comparisons. Cytokine data were available for a subgroup of 30 patients (observation *N* = 18, control *N* = 12). IQR, interquartile range. *P < 0.05; **P < 0.01

### Result 5. There was no difference in levels of immunoglobulins and complements between the two groups

Comparative analysis of humoral immunity parameters revealed no significant differences between anti-M positive patients and controls across immunoglobulin profiles (IgE: 46.1 vs. 30.3 IU/mL, *P* = 0.179; IgG: 9.65 vs. 7.99 g/L, *P* = 0.322; IgA: 0.95 vs. 0.77 g/L, *P* = 0.399; IgM: 0.90 vs. 0.84 g/L, *P* = 0.160) and complement components (C3: 1.10 vs. 1.09 g/L, *P* = 0.695; C4: 0.23 vs. 0.27 g/L, *P* = 0.734) (Table 5). This indicates that anti-M antibody development in this cohort neither resulted from nor induced generalized hypergammaglobulinemia or complement consumption. The immune response demonstrated strict antigenic specificity against the M epitope, showing no evidence of broad B-cell hyperactivity or immunodeficiency.

**Table 5:** Comparison of immunoglobulins and complements between groups

Characteristics	Control (*N* = 31)	Observation (*N* = 31)	*P* value
IgE (IU/mL), Median (IQR)	30.3 (37.3)	46.1 (119.6)	0.179
IgG (g/L), Median (IQR)	7.99 (4.08)	9.65 (4.14)	0.322
IgA (g/L), Median (IQR)	0.77 (0.59)	0.95 (1.39)	0.399
IgM (g/L), Median (IQR)	0.84 (0.37)	0.90 (0.57)	0.160
C3 (g/L), Median (IQR)	1.09 (0.31)	1.10 (0.20)	0.695
C4 (g/L), Median (IQR)	0.27 (0.17)	0.23 (0.16)	0.734

Values are presented as median (IQR). Mann-Whitney U test was used for all comparisons. IQR, interquartile range

## Discussion

The association between microbial infections and naturally occurring anti-M antibodies has been recognized for decades. Kao et al. first demonstrated that children with acute bacterial infections caused by *Haemophilus influenzae*, *Proteus mirabilis*, *Staphylococcus aureus*, and *Neisseria meningitidis* could develop anti-M antibodies [6]. Our study extends these observations to SARS-CoV-2, demonstrating that COVID-19 infection within 2 years was present in 54.84% of anti-M positive children compared to only 19.35% of matched controls (OR = 5.06, *P* = 0.008). The temporal relationship between COVID-19 infection and anti-M antibody titers provides additional support for an association: we observed a significant inverse correlation between the interval since COVID-19 infection and IgG anti-M titers (*r* = -0.63, *P* = 0.008), indicating that more recent infection was associated with higher antibody levels. Our findings align with the case report by Jeannet et al., who described de novo anti-M emergence in a severe COVID-19 patient and identified an unmutated IgM lambda clone through RACE-repertoire sequencing [10]. However, our study is the first to investigate this association in a pediatric cohort with appropriate controls. The mechanism likely involves molecular mimicry between SARS-CoV-2 surface glycoproteins and the M antigen epitope on glycophorin A (GPA). The spike protein of SARS-CoV-2 is heavily glycosylated [12], and structural similarities with GPA epitopes may drive cross-reactive B cell activation. In severe COVID-19, impaired T-B cell collaboration can lead to extrafollicular B cell activation, producing low-affinity antibodies without class switching [9]. However, the non-critically ill children in our cohort developed higher-affinity class-switched antibodies, suggesting prolonged germinal center reactions. Notably, 22.58% (7/31) of our anti-M positive children had tonsillar and/or adenoid hypertrophy, and SARS-CoV-2-specific germinal center B cells have been shown to persist in pediatric tonsils and adenoids, potentially facilitating affinity maturation toward pathogenic IgG subclasses [13].

Our serological characterization revealed important findings regarding the immunological properties of pediatric anti-M antibodies. In this cohort, IgG-class antibodies constituted the dominant response (80.65%, 25/31), with 32.26% being IgG-only, 48.39% IgG + IgM, and only 19.35% IgM-only. This distribution differs substantially from previous reports in adult populations: Wang et al. found that IgM + IgG was the dominant profile in Chinese adults (71%), with IgG-only antibodies being exceptionally rare (1%) [14], whereas our pediatric data showed 32-fold higher prevalence (32.26%). Several factors may contribute to this difference: pediatric B cell repertoires are enriched for naive clonal phenotypes with distinct selection dynamics, potentially facilitating more efficient class switching [15]; children experience more frequent respiratory infections creating repeated opportunities for cross-reactive B cell activation through molecular mimicry; and the unique mucosal immune environment in children, particularly nasopharynx-associated lymphoid tissue, provides specialized niches for sustained germinal center reactions [13]. These features suggest that pediatric anti-M antibodies may represent a distinct entity from adult naturally occurring antibodies, with potentially different clinical implications.

Most significantly, we observed that reactive IgG antibodies (IgG1 and/or IgG3 positive) exhibited dramatically higher titers than inactive IgG (median 64 vs. 4, *P* < 0.001), representing a 16-fold increase. All high-titer cases (IgG ≥ 64, *n* = 11, 35.48%) were exclusively confined to the reactive IgG group, with no high-titer cases in the inactive IgG group. Since IgG1 and IgG3 subclasses potently activate complement and Fc-receptor-mediated effector functions including phagocytosis and antibody-dependent cellular cytotoxicity (ADCC) [16, 17], this finding carries important clinical relevance: over one-third of anti-M positive children had antibodies capable of causing hemolytic transfusion reactions or hemolytic disease of the fetus and newborn (HDFN). Crucially, the absence of high-titer (≥ 64) IgG antibodies in the inactive IgG group underscores the necessity of IgG subclass determination for effective clinical risk stratification. The significantly elevated plasma levels of IL-8 and IL-12p70 in anti-M positive patients revealed a distinct inflammatory signature associated with anti-M antibody production. IL-8 is a potent neutrophil chemoattractant and a hallmark of pediatric COVID-19 [18], while IL-12p70 promotes Th1 polarization and IFN-γ production [19]. While inflammation is known to play a central role in red blood cell alloimmunization [20], the specific cytokine profile associated with infection-triggered anti-M antibodies has not been previously characterized. Our findings provide the first description of this inflammatory signature. Within this milieu, IL-12p70 may enhance B-cell activation and antibody production, while IL-8-driven neutrophilic responses could alter antigen presentation, potentially lowering the threshold for cross-reactive B-cell activation against the M antigen and facilitating isotype switching to pathogenic IgG1/IgG3 subclasses. Whether this profile is specific to COVID-19-associated anti-M or represents a general feature of infection-induced anti-M remains to be determined.

The absence of significant differences in systemic immunoglobulin (IgE, IgG, IgA, IgM) or complement (C3 and C4) levels between groups indicates that anti-M antibody development demonstrated strict antigenic specificity without generalized humoral dysregulation, further supporting the role of targeted triggers such as molecular mimicry. Notably, anti-M positive patients had significantly lower bilirubin levels despite the presence of potentially pathogenic antibodies, providing reassurance that these antibodies were not causing clinically significant hemolysis at the time of detection. This may involve infection-related enhancement of hepatobiliary bilirubin excretion [21] and IL-8-mediated suppression of bilirubin synthesis [22], rather than increased hemolysis. Similarly, the higher platelet count in the observation group (*P* = 0.043) may reflect enhanced thrombopoietic activity associated with infection and immune activation [23, 24].

Our findings have direct implications for transfusion practice. Over one-third (35.48%) of anti-M positive children had high-titer reactive IgG antibodies that could potentially cause hemolytic reactions if transfused with M-antigen positive blood. As COVID-19 infections continue to occur in periodic outbreaks, it is crucial to enhance screening for unexpected antibodies such as anti-M, especially for pediatric patients requiring blood transfusions. Given the association with COVID-19 and other viral infections, we suggest the following considerations for clinical practice, which require prospective validation: (1) clinicians should be aware that children with recent COVID-19 or other viral infections may have a higher likelihood of developing anti-M antibodies, supporting the continued importance of routine pre-transfusion antibody screening; (2) when anti-M antibodies are detected, IgG subclass determination (IgG1/IgG3) may provide additional risk stratification information, as our data demonstrate that all high-titer cases were exclusively in the reactive IgG group; and [3] for patients identified with high-titer reactive IgG anti-M (≥ 64, IgG1/IgG3 positive), provision of M-antigen negative blood should be considered, consistent with established transfusion medicine practice for clinically significant alloantibodies. In settings where IgG subclass testing is not readily available, IgG titer determination alone can serve as a surrogate, given the strong correlation between reactive IgG status and high titers (median 64 vs. 4, *P* < 0.001).

Based on our findings, we propose a preliminary risk stratification for pediatric patients with anti-M antibodies: High risk, reactive IgG (IgG1/IgG3 positive) with titer ≥ 64, requiring M-antigen negative blood; Moderate risk, reactive IgG with titer < 64, requiring compatibility testing and close monitoring; Low risk, inactive IgG (IgG1/IgG3 negative) or IgM-only, where standard compatibility procedures may suffice. This stratification requires prospective validation before clinical implementation.

### Limitations

Several limitations should be acknowledged. First, this is a single-center retrospective study with a modest sample size (*N* = 31 per group). Post-hoc power analysis indicated 82.4% power for the primary outcome (COVID-19 - anti-M association, Cohen’s h = 0.76), but the study was underpowered for detecting smaller effects, particularly in the cytokine subgroup (*N* = 30). Multi-center prospective studies with larger sample sizes are essential to validate our findings. Second, cytokine data were available for only a subgroup of 30 patients (observation *N* = 18, control *N* = 12) due to residual plasma volume limitations. Although baseline characteristics did not differ significantly between cytokine-tested and non-tested subgroups (Supplementary Table S1), the reduced sample size limits the reliability of cytokine findings. Third, we could not completely exclude the confounding effect of concurrent infections, as anti-M positive patients had higher rates of other pathogen infections; however, COVID-19 infection history was independently assessed based on reported records within 2 years, providing a distinct time window from current pathogen status. Fourth, we lacked baseline samples before COVID-19 infection to definitively establish temporal causality. While the significant association (OR = 5.06), the dose-response pattern (*r* = -0.63), and the biological plausibility of molecular mimicry collectively support an infection-triggered mechanism, we cannot exclude reverse causation or unmeasured confounding. The molecular mimicry hypothesis requires direct experimental validation through structural epitope comparison or in vitro cross-reactivity assays. Fifth, COVID-19 infection history was ascertained through telephone follow-up interviews with parents/guardians rather than direct medical documentation, which may be subject to recall bias. Undocumented asymptomatic infections in the control group could lead to non-differential misclassification, potentially attenuating the observed association. Additionally, our study did not systematically collect COVID-19 vaccination history, as pediatric vaccinations in China were primarily administered at community health service centers, and no centralized cross-institutional vaccination database was available for retrospective retrieval. Future prospective studies should document vaccination status and timing to better delineate their respective contributions to anti-M antibody development.

## Conclusion

This study provides evidence of a significant association between COVID-19 infection and anti-M alloantibody development in NN phenotype pediatric patients. Over one-third of affected children developed high-titer reactive IgG antibodies with potential clinical significance for transfusion safety. Anti-M positive patients exhibited a distinct inflammatory profile characterized by elevated IL-8 and IL-12p70, without evidence of global humoral dysregulation. These findings suggest the need for enhanced antibody screening in pediatric transfusion candidates with recent viral infections and highlight the potential value of IgG subclass determination for risk stratification, pending validation in larger multi-center prospective studies.

## Electronic Supplementary Material

Below is the link to the electronic supplementary material.

Supplementary Material 1

## Data Availability

The data used to support the findings of this study are available from the corresponding author upon request.

## Abbreviations

AHG: Anti-Human Globulin
C3: Complement Component 3
C4: Complement Component 4
CI: Confidence Interval
COVID-19: Coronavirus Disease 2019
CRP: C-Reactive Protein
DTT: Dithiothreitol
EDTA: Ethylenediaminetetraacetic Acid
GPA: Glycophorin A
HDFN: Hemolytic Disease of the Fetus and Newborn
IAT: Indirect Antiglobulin Test
IFN-α: Interferon Alpha
IFN-γ: Interferon Gamma
IgA: Immunoglobulin A
IgE: Immunoglobulin E
IgG: Immunoglobulin G
IgM: Immunoglobulin M
IL-1β: Interleukin-1 Beta
IL-2: Interleukin-2
IL-4: Interleukin-4
IL-5: Interleukin-5
IL-6: Interleukin-6
IL-8: Interleukin-8
IL-10: Interleukin-10
IL-12p70: Interleukin-12 p70
IL-17: Interleukin-17
IQR: Interquartile Range
MFI: Mean Fluorescence Intensity
MNS: MNS Blood Group System
OR: Odds Ratio
PE: Phycoerythrin
PSM: Propensity Score Matching
RBC: Red Blood Cell
SARS-CoV-2: Severe Acute Respiratory Syndrome Coronavirus 2
TNF-α: Tumor Necrosis Factor Alpha
WBC: White Blood Cell
