# Supplementary material for: COVID-19 infection is associated with anti-M alloantibody development in pediatric patients: immunological characteristics and clinical implications for transfusion safety
Source: Ital J Pediatr. 2026 Apr 24;52:100. doi: 10.1186/s13052-026-02258-x (PMC13255407; doi:10.1186/s13052-026-02258-x)
Supplement: Supplementary file 1 — Supplementary Material 1 [file 13052_2026_2258_MOESM1_ESM.docx]

**Supplementary Table S1. Comparison of Baseline Characteristics Between Cytokine-Tested and Non-Tested Subgroups**

| **Characteristics** | **Observation Group** | | | **Control Group** | | |
| --- | --- | --- | --- | --- | --- | --- |
|  | **Tested (N = 18)** | **Non-tested (N = 13)** | **P value** | **Tested (N = 12)** | **Non-tested (N = 19)** | **P value** |
| Age (months), Median (IQR) | 42.0 (63.8) | 35.0 (40.0) | 0.298 | 49.5 (52.5) | 32.0 (52.5) | 0.282 |
| Male, N (%) | 9 (50.0) | 7 (53.8) | 1.000 | 6 (50.0) | 12 (63.2) | 0.710 |
| COVID-19 Positive, N (%) | 9 (50.0) | 8 (61.5) | 0.717 | 4 (33.3) | 2 (10.5) | 0.174 |

Continuous variables are presented as median (IQR) and compared using Mann-Whitney U test; categorical variables are presented as N (%) and compared using Fisher’s exact test. IQR, interquartile range.
